# Antimicrobial Mechanisms and Clinical Application Prospects of Antimicrobial Peptides

**DOI:** 10.3390/molecules27092675

**Published:** 2022-04-21

**Authors:** Xin Li, Siyao Zuo, Bin Wang, Kaiyu Zhang, Yang Wang

**Affiliations:** 1 Department of Infectious Diseases, First Hospital of Jilin University, Changchun 130021, China; lixinlixingogo@jlu.edu.cn (X.L.); wangbinlucky2018@jlu.edu.cn (B.W.); 2 Department of Dermatology and Venereology, First Hospital of Jilin University, Changchun 130021, China; zuosy17@jlu.edu.cn

**Keywords:** antimicrobial peptides, structural characteristics, antimicrobial mechanism, clinical application

## Abstract

Antimicrobial peptides are a type of small-molecule peptide that widely exist in nature and are components of the innate immunity of almost all living things. They play an important role in resisting foreign invading microorganisms. Antimicrobial peptides have a wide range of antibacterial activities against bacteria, fungi, viruses and other microorganisms. They are active against traditional antibiotic-resistant strains and do not easily induce the development of drug resistance. Therefore, they have become a hot spot of medical research and are expected to become a new substitute for fighting microbial infection and represent a new method for treating drug-resistant bacteria. This review briefly introduces the source and structural characteristics of antimicrobial peptides and describes those that have been used against common clinical microorganisms (bacteria, fungi, viruses, and especially coronaviruses), focusing on their antimicrobial mechanism of action and clinical application prospects.

## 1. Introduction

Antimicrobial peptides (AMPs) are small molecular peptides (generally composed of less than 100 amino acid residues) [1] that widely exist in nature. They can be extracted from bacteria, fungi, plants, insects, amphibians, fish, birds, mammals and even the human body. AMPs are an important part of almost all biological innate immunity and play an important role in resisting the invasion of foreign microorganisms [2]. AMPs usually have a positive net charge and an amphiphilic structure that allows strong interactions with hydrophobic surfaces and membranes [1,3], showing strong broad-spectrum activity against bacteria, fungi, viruses, and other microorganisms [3,4,5]. AMPs also exhibit antimicrobial activity by targeting intracellular targets to inhibit the synthesis of cell walls, nucleic acids, and proteins and regulate host immune responses [6,7]. Pathogen resistance is increasing at an unprecedented rate and has become a major global public health threat [1,3]. The original antimicrobial mechanisms of AMPs can better solve the problem of the increasing resistance of pathogenic microorganisms to antibiotics [8], which has aroused great interest from researchers in the field of biomedicine. In addition, AMPs are being actively researched and developed as an alternative therapy for viral infections, such as coronavirus disease 2019 (COVID-19) and human immunodeficiency virus (HIV) infection [9,10]. Since microbiologist René Dubos discovered gramicidin, the first tested antimicrobial agent isolated from soil Bacillus strains in 1939, more than 3000 AMPs have been reported in the AMP database 3(APD3), the antimicrobial peptide database, which is a tool for research and education [11].

Next, we briefly introduce the source, structural characteristics and biological activity of AMPs and elaborate on AMPs with antimicrobial activity against common bacteria, fungi and viruses, their mechanism of action, and their clinical applications.

## 2. Sources, Structures and Activities of Antimicrobial Peptides

### 2.1. Sources of AMPs

AMPs widely exist in various species, including microorganisms, animals, plants and even marine organisms [11]. Next, we briefly introduce the representative AMPs of each species.

#### 2.1.1. AMPs from Microorganisms

Polymyxins from the genus Bacillus are a group of cyclic peptide antibiotics produced by *Bacillus polymyxa* [12]. Among them, polymyxin B and polymyxin E (colistin) have been commercialized and applied in clinical practice, showing good effects against multidrug-resistant Gram-negative bacilli [13,14]. Although the widespread clinical use of these drugs has been limited in the past due to their renal and neural toxicities, the continuous optimization of formulation, reduction in drug dose, avoidance of drug combinations with other potential renal/neural toxic drugs, as well as comprehensive and better care and possibly better manufacturing, have reduced the incidence of drug side effects [14,15]. Sumi et al. have well summarized the AMPs derived from the genus *Bacillus* [16]. Nisin is a natural bioactive AMP with strong bactericidal activity synthesized and secreted by *Lactococcus* and *Streptococcus* in the process of metabolism. It has antibacterial activity against many Gram-positive cocci. It also has a certain inhibitory effect on Gram-negative bacilli under the action of a complexing agent or fusion with a short peptide with anti-Gram-negative bacterial activity [17]. As a biological preservative, it is widely used in the food industry. It also has a variety of applications in the biomedical field, including for bacterial infection, cancer, oral diseases and other fields, with recognized clinical application potential [18]. Besides, there are many AMPs derived from different bacteria, such as bacitracin produced by *Bacillus* [19], gramicidin S produced by *Bacillus brevis* [20] and vancomycin produced by *Streptococcus orientalis* [21]. Fungal defensins, such as triinsin and bldesin, have been extracted from fungi and identified as having antimicrobial activity [22,23]. There are also many types of AMPs produced by fungi, such as amatoxins and phallotoxins produced by *Amanita phalloides* mushrooms [24], and dikaritins produced by *Ascomycetes* [25]. In conclusion, AMPs produced by microorganisms are widely diverse.

#### 2.1.2. AMPs from Animals and Plants

The well-known families of AMPs derived from mammals are the cathelicidin family and the defensin family. Nearly 30 different cathelicidin types have been described in different kinds of mammals. However, LL-37 is the only AMP in the cathelicidin family that exists in the human body, produced mainly by epithelial cells and neutrophils [26]. In addition to lipopolysaccharide (LPS) neutralization and antibacterial activity, LL-37 also has many different biological activities, such as the regulation of inflammation. It has been verified that LL-37 can protect the body through a variety of mechanisms in a murine sepsis model, such as the modulation of cell death and the release of antimicrobial neutrophil extracellular traps and ectosomes, as well as direct bactericidal and LPS-neutralizing activities [26]. In addition, LL-37 has potential value in preventing and treating drug-resistant bacterial infections and inhibiting biofilm formation [27]. Defensins are the primary components of the animal defense system. Most of them consist of 29~42 amino acid residues and contain three pairs of intramolecular disulfide bonds with a relative molecular weight of 2~6 ku. According to the position of disulfide bonds, defensins can be divided into three classes: α-defensins, β-defensins and θ-defensins. Humans do not express θ-defensins [28]. Host defense peptides (HDPs) can be detected in many parts of the body, such as the skin, eyes, ears, mouth, respiratory tract, lungs, intestines, and urethra. Some AMPs are constitutively produced, while the production of others is induced during infection and inflammation/injury [29]. The types of AMPs expressed also vary at different growth stages in humans, such as LL-37, which is usually detected in the skin of newborn infants, and human β-defensin 2 (HBD-2), which is usually expressed in elderly individuals rather than in younger individuals [30]. The constitutive expression of HBD-2, HBD-3 and cathelicidin has been shown to be significantly higher in keratinocytes from fetal skin than in keratinocytes from postnatal skin [30].

Frogs are the main source of amphibian AMPs. The most famous AMPs in frogs are magainins, which were first isolated from the skin of the African clawed frog *Xenopus laevis*. Studies have shown that magainins have antibacterial activity against a large number of Gram-positive and Gram-negative bacteria and some fungi from the human body [31]. Studies have also shown that magainin 2 has synergistic antibacterial and pore formation activities with another positively charged and amphipathic AMP, PGLa, that is mediated by anchoring between the anionic C-terminus of magainin 2 and the cationic C-terminus of PGLa [32,33].

Insects are also major sources of AMPs, and cecropins are the best known AMP family from insects, which were first isolated from the hemolymph (insect blood) of *Hyalophora cecropia* pupae and were characterized for their antimicrobial activity against several Gram-positive and Gram-negative bacteria, as well as fungi [34,35,36]. Subsequently, these peptides have been identified in two other orders of *Coleoptera* and *Diptera*, as well as in other species of *Lepidoptera* [37,38]. Other insect AMPs, such as insect defensins, proline-rich peptides and attacins, have also been found and well studied [37]. The classification, structure and potential applications of insect AMPs are well summarized in the review by Yi et al. [37].

AMPs are widely distributed in plants. A large number of AMPs can be extracted and isolated from plants and plant organs, such as stems, roots, seeds, flowers and leaves. According to their amino acid sequence and structure, they can be divided into several categories, including thionins, defensins, snakins, lipid transfer proteins, glycine-rich proteins, cyclotides and hevein-type proteins [39]. They perform various physiological defensive mechanisms to eliminate viruses, bacteria, fungi and parasites and can be used as therapeutic and preservative agents [40].

#### 2.1.3. AMPs from Marine Organisms

Most antibiotics come from terrestrial ecosystem constituents: fungi, soil-borne bacteria, and some plants. There are an increasing number of studies on AMPs in marine organisms [11,41]. A recent in vitro study on the antibacterial potential of a peptide extract from the marine mollusk *Olivacillaria hiatula* showed that the peptide extract had an inhibitory effect on both Gram-positive and Gram-negative bacteria [41]. Another antibacterial peptide, MFAP9, isolated and purified from marine *Aspergillus fumigatus* BTMF9, exhibited strong antibacterial activity against *Bacillus circulans*, had a strong antibiofilm effect, and was nontoxic to human red blood cells at the experimental concentrations. MFAP9 is a new type of anti-infective drug suitable for development [42]. Polyphemusin-I is a marine AMP obtained from hemocyte debris of *Lumulus polyphemus* (American horse-shoe crab), possesses high antibacterial activity against a variety of pathogenic microorganisms, such as *Escherichia coli* and *Candida albicans* [43]. AaCrus1, a novel marine AMP identified from *Amphibalanus amphitrite*, has antibacterial activity against a variety of Gram-positive and Gram-negative bacteria [44].

### 2.2. Structures and Activities of AMPs

There are many types of AMPs, which are roughly divided into four categories according to their secondary structure, including α-helical AMPs, β-sheet-containing AMPs, AMPs with a loop stabilized by a single disulfide bond or cyclization of the peptide chain and short AMPs with extended conformations [45]. Some AMPs consist entirely of an α-helix or β-sheet, while others have a more complex structure. Among them, α-helical peptides are the most studied AMPs thus far, such as magainin [46] and LL-37 [47]. The main characteristics of this class of AMPs are that they do not contain cysteine, do not form intramolecular disulfide bonds, and are disordered in aqueous solutions but form amphiphilic helical structures in membranes or membrane-simulated environments [48]. α-helical AMPs are widely distributed, are abundant in quantity, have broad-spectrum antimicrobial properties, are short in length (generally <40 amino acid residues) and are easy to chemically synthesize. Furthermore, these AMPs have a simple linear structure, can be easily structurally characterized and have strong antimicrobial activity. They have few side effects on mammalian cells. Therefore, the structure of α-helical peptides is currently a primary research focus. β-sheet-containing AMPs have an anti-parallel β-sheet structure, containing 2-4 disulfide bonds in the molecule. Disulfide bonds exist between two peptides that constitute β-sheets to stabilize the AMP structure and promote penetration the cell membrane [48]. Representative AMPs are α- and β-defensins [28], drosomycin [49], and plectasin [50]. Cyclic AMPs include peptides with a cyclic structure formed by one or more disulfide bonds, including bactenecin [51], subtilosin A [52], polymyxin B [12], and θ-defensins [53]. The extended AMPs lack a typical secondary structure and are rich in specific amino acids, such as proline, tryptophan, arginine, glycine and histidine. Their antimicrobial activity is exerted by hydrogen bonds or van der Waals forces between peptides and membrane lipids, independent of hydrogen bonds between residues. Most extended AMPs achieve antimicrobial activity by penetrating the cell membrane of pathogens and interacting with intracellular targets [48]. Extended AMPs include proline-rich peptides, such as insect oncocins and bovine bactenecins [54]; tryptophan-rich peptides, such as indolicidin, tritrpticin and lactoferricin [55]; and histidine-rich peptides, such as histatin-3 and histatin-5 [56].

The activity of AMPs is related to many factors, including the size, sequence, charge, structure and conformation, hydrophobicity and amphipathic properties. Their biological functions can be divided into the following aspects. (1) Direct bactericidal effect: AMPs directly kill microorganisms by acting on the microbial membrane or intracellular targets. Most AMPs have direct bactericidal activity, such as LL-37 [57] and B22 [6]; (2) Antibiofilm activity: A number of studies have shown that AMPs have antibiofilm activity, whether the concentration is equal to or higher than the minimum inhibitory concentration (MIC) for corresponding planktonic cells or the concentration is lower than the MIC for corresponding planktonic cells [2,58]; (3) Immune system regulation: This mechanism includes regulation of proinflammatory and anti-inflammatory responses, chemotaxis, cell differentiation, wound healing, autophagy, apoptosis and pyrolysis and enhancement of host immune cell activity. For example, the host defense peptides LL-32 and polymyxin B can neutralize endotoxins by multifactorial mechanisms, including LPS interaction and targeting of host cell membranes [59]. LL-37 can not only neutralize proinflammatory factors by inhibiting endotoxin activity but also protect the body from serious damage and complications by inhibiting pyrolysis [26]; (4) When used in combination with traditional antibiotics, AMPs also show a synergistic effect by promoting the absorption of antibiotics [60]; (5) Some AMPs also have anticancer effects. For example, epinecidin-1 is a cationic AMP with an α-helical structure. Studies have reported seven functional uses of the peptide, including those based on its antibacterial, antifungal, antiviral, antiprotozoal, anticancer, immune regulation and wound healing properties [61].

## 3. Mechanism of Action of Antimicrobial Peptides

The antimicrobial activity of AMPs is achieved through complex mechanisms of action, including acting on the cell wall, cell membrane, and different intracellular targets, as well as antibiofilm formation and host immune system modulation activities. We will introduce them one by one.

### 3.1. AMPs Acting on the Cell Wall

**Figure 1:**
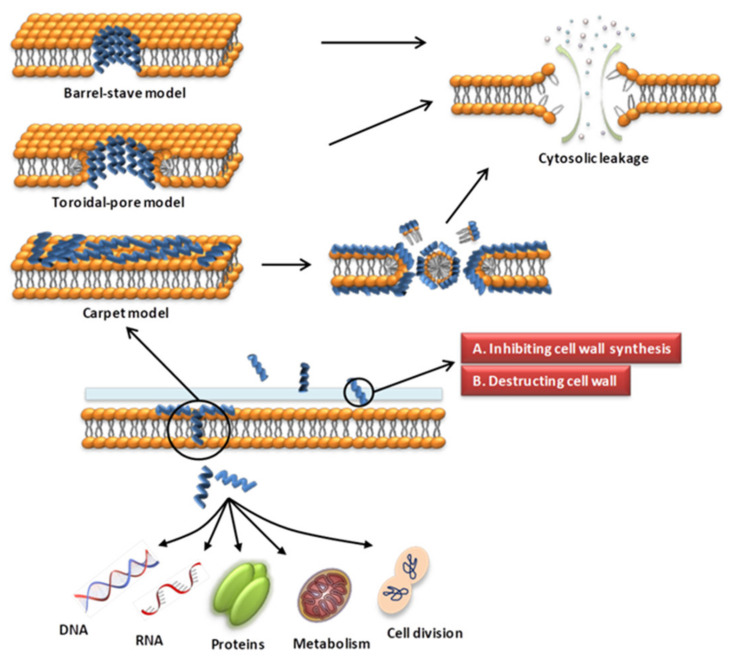
Schematic presentation of the antibacterial mechanism of AMPs. Some AMPs act on cell membranes through different modes of action, increasing membrane permeability, leading to leakage of cell contents and cell death. Modes of action include barrel-stave, toroidal-pore, carpet models. Some AMPs act on the cell wall and exert antibacterial effects by affecting the synthesis of cell wall components and destroying the cell wall structure. Some AMPs enter the cell through direct penetration or endocytosis, and exert anti-microbial effects by targeting the nucleus, organelles, present in fungi, or intracellular proteins.

The cell wall of bacteria is essential for their survival [62,63]. The cell wall of bacteria is the outermost layer of the cell surrounding the cell membrane. It is a membrane-like structure with a complex composition that varies among different bacteria. Gram-positive bacteria have thicker cell walls, 15–50 layers of peptidoglycan (PGN), and a large amount of teichoic acid, which can be classified as wall teichoic acid (WTA) or membrane teichoic acid [64,65]. Gram-negative bacteria have thin cell walls and complex structures, containing 1–2 layers of PGN and a unique outer membrane, which is composed of lipoproteins, a lipid bilayer and LPS [64,65]. Fungal cell walls are mainly composed of glucan, chitin and glycoproteins [66]. The cell wall is a potential target for AMPs to recognize microbial cells. AMPs acting on the cell wall exert antibacterial effects mainly by affecting the synthesis of cell wall components and destroying the cell wall structure (Figure 1). The specific mechanisms of action are as follows.

(1) AMPs interfere with the biosynthesis of PGN, a component of the cell wall, thereby causing damage to the cell wall. The bacterial PGN layer is critical to bacterial integrity and survival and is a major target of many antibiotics. Lipid II is an important component in the synthesis of PGN, which is located on the cytoplasmic side of the cell membrane and transports cell wall subunits across the cell membrane for polymerization and insertion into the existing cell wall [67]. AMPs can bind to the cell wall synthesis precursor lipid II, which in turn interferes with further enzymatic processes, thereby inhibiting PGN synthesis [65]. Representative AMPs include vancomycin [68] and oritavancin [69], which bind to the alanine stem and pentaacyl bridge of the pentapeptide portion of lipid II and inhibit the synthesis of PGN by inhibiting transglycosylation and transpeptidation [69]. Nisin also targets lipid II to exert antibacterial effects, and nisin additionally recruits lipid II to form pores through the plasma membrane [70]. Teixobactin, a depsipeptide, arrests cell wall synthesis by selectively inhibiting the transglycosylation of PGN [71,72]. Other AMPs targeting lipid II include plectasin [73], plusbacin-A3 (pb-A3) [74], and tridecaptin A1 [75]. Ramoplanin, a lipoglycopeptide antibiotic, inhibits the biosynthesis of Gram-positive bacterial cell wall PGN by inhibiting the conversion of lipid intermediate I to lipid intermediate II catalyzed by N-acetylglucosamine transferase [76]. Colicin M was demonstrated to provoke *Escherichia coli* cell lysis via inhibition of cell wall PGN biosynthesis by enzymatic degradation of the undecaprenyl phosphate-linked PGN precursors [77];

(2) AMPs damage cell wall integrity by inhibiting the biosynthesis of WTA. For example, in addition to inhibiting the biosynthesis of PGN precursors lipid I and lipid II, teixobactin can simultaneously inhibit the biosynthesis of teichoic acid lipid III, the main precursor of WTA, thereby exerting a bactericidal effect on methicillin-resistant *Staphylococcus aureus* (MRSA) [72];

(3) Some AMPs destroy bacterial cell walls by releasing autolysins. Pep5 and nisin are cationic polypeptide antibiotics, and in addition to their membrane-disrupting effects, they can also induce the autolysis of staphylococci. Pep5 and nisin show high affinity for the phosphate wall and teichuronic acid of Gram-positive bacterial cell walls and competitively displace cell wall-associated amidases, which results in the premature release of autolysins, leading to cell lysis [78];

(4) The outer membrane is a special component of the cell wall of Gram-negative bacteria, and AMPs acting on the outer membrane can exert anti-Gram-negative bacterial activity. For example, thanatin, an insect-derived AMP, can act on a variety of microorganisms through multiple modes of action. One of the modes of action is to induce the aggregation of LPS micelles, disrupt the outer membrane of Gram-negative bacteria, and cause outer membrane charge neutralization. In addition, the binding of thanatin to the outer membrane can competitively replace the Ca2+ ions stably bound to LPS molecules in the outer membrane and induce the release of LPS, thereby destroying the outer membrane of bacteria [79]. Many studies have demonstrated that thanatin’s activity against Gram-negative cells occurs mainly through the inhibition of LPS export in combination with lipopolysaccharide transport protein A (LptA), a crucial component of the LPS export machinery [80];

(5) In fungi, some AMPs exert antifungal effects by inhibiting the synthesis of fungal cell wall components, such as glucan, chitin, and glycoproteins. For example, the glucan inhibitor echinocandin is a noncompetitive inhibitor of β-(1,3)-glucan synthase, which affects fungal cell wall synthesis. Representative AMPs are caspofungin, micafungin and anidulafungin [63]. The cysteine-reduced form of psoriasin, as a fungicidal AMP, binds to β-glucan, a basic component of the *Candida albicans* cell wall, thereby inhibiting the adhesion of the pathogen to surfaces and increasing IL-8 production by mucosal epithelial cells [81]. Nikkomycin Z, a dipeptide with a nucleoside sidechain synthesized by *Streptomyces tendae*, is a competitive inhibitor of chitin synthases [82]. Pradimicins and benanomicins target cell wall mannan [83]. These antifungal peptides (AFPs) have been well summarized in a previous review [63]. AMP-17 is a novel antibacterial peptide from houseflies with good antifungal effects against *Candida*. Morphological observation showed that AMP-17 disrupted the integrity of the *C. albicans* cell wall. After AMP-17 treatment, the expression of the *C. albicans* cell wall synthesis-related gene FKS2 was upregulated in response to AMP-17-induced fungal cell wall damage [84]. In addition, AMP-17 can also destroy the *Candida* cell membrane by downregulating the expression of ergosterol synthesis-related genes, reducing their gene product levels in the cell membrane [84];

(6) Some AMPs interact with cell wall components to mediate agglutination and capture of microorganisms. Amyloid-β peptide (Aβ), a key protein in Alzheimer’s disease (AD) pathology, is an AMP. Its mechanism of action is that Aβ oligomers first bind to microbial cell wall carbohydrates through the heparin-binding domain, and propagating β-amyloid fibrils mediates the agglutination and eventual capture of unattached microorganisms, inhibiting pathogen adherence to host cells [85]. Thanatin can bind to outer membrane LPS and form micellar complexes with LPS, leading to cell aggregation or agglutination [86].

### 3.2. AMPs Acting on the Cell Membrane

The microbial cell membrane is an important target of most AMPs, and the difference from the mammalian cell membrane composition is a major factor in the selective killing of bacteria by AMPs [87]. AMPs combine with the microbial cell membrane through physicochemical action, and continuously accumulate on the surface of the cell membrane and undergo structural or conformational transitions. When a certain threshold concentration is reached, AMPs can act on different microorganisms through different modes of action, increasing the permeability of the membrane and leading to lysis of cell membranes and release of cellular contents, thereby exerting antibacterial activity [87,88].

In the process of AMPs interacting with cell membranes, there are two main factors affecting the interaction, namely, conformational changes and the peptide-lipid ratio [87,89,90]. Studies have shown that α-helical AMPs have a disordered structure in an aqueous environment; however, upon binding to a phospholipid bilayer, they rapidly form a strictly amphiphilic α-helical conformation that facilitates the interaction with the membranes [91]. A study on the effect of KLA polypeptide conformation and membrane surface properties on the electrostatic binding process showed that KLA polypeptides with low helical propensity tended to form β structures at low lipid-peptide ratios [92]. Helical KLA polypeptides stabilize the anionic gel-state lipid 1,2-dipalmitoyl-sn-glycero-3-phosphoglycerol (DPPG) bilayer, while β-structured polypeptides cause significant membrane perturbations [92]. The binding efficiency of helical KLA peptides to DPPG vesicles has been shown to be higher than that of β-structured KLA peptides, and the binding affinity was proportional to the helical tendency of the peptides and the negative charge on the membrane surface [92]. Another study showed that KLA peptides are flexible and highly dynamic in adapting to their surroundings by adopting different conformations [92]. The peptide-lipid ratio is another major factor affecting the interaction of AMPs with cell membranes. When the peptide-lipid ratio is low, AMPs are parallel to the surface of the plasma membrane, and as the peptide-lipid ratio increases, AMPs directly insert into the hydrophobic center of the membrane [93,94]. Ultimately, membrane permeability is increased, leading to cell death.

Various modes of AMP interaction with cell membranes have been proposed. The three models most commonly discussed are the barrel-stave, toroidal-pore, and carpet models (Figure 1). They have been addressed in several literature reviews [63,87,88]. We only briefly introduce them here. (1) In the barrel-stave model, helical polypeptides aggregate within the cell wall and are introduced vertically into the lipid bilayer to form bundle-like pores in the membrane, thereby affecting the permeability of the membrane [95] (Figure 1). Only a few AMPs, such as alamethicin [96,97], ceratotoxins [98] and protegrins [99], have been shown to form barrel stave channels. (2) In the toroidal-pore model, peptides interact with the lipid head groups, inducing the bilayer to bend and vertically insert into the membrane bilayer and form annular holes composed of the peptides and phospholipid head bundles [100] (Figure 1). Representative AMPs are magainins [101], melittin [96], protegrins [96,102], actinoporins [103] and lacticin Q [100]. (3) In the carpet model, AMPs cover the membrane surface in a carpet shape and interact with the membrane in parallel through electrostatic interaction with the anionic phospholipid head groups. The formation of micelles at high peptide concentrations damages the phospholipid bilayer [93,104] (Figure 1). Magainin [104], Citropin 1.1 [105] and LL-37 [106,107] are representative AMPs that disrupt the cytoplasmic membrane via the carpet model.

Some AMPs interact with cell membranes through different modes of action, depending on the source and lipid composition of the membrane [104]. Since most amphiphilic AMPs have an overall positive charge, cell membranes with large amounts of anionic phospholipids, such as phosphatidylserine and phosphatidylglycerol, may be more susceptible to AMPs [104]. A study determined that the curvature strain of the lipid bilayer significantly affects how magainin interacts with the lipid bilayer [108]. With the addition of phosphatidylethanolamine, a zwitterionic phospholipid, the curvature strain of the lipid bilayer becomes more negative, and magainin fails to form annular pores [108]. In contrast, the peptide disrupts the bilayer via the carpet model [108].

In addition, there are receptor-mediated AMPs that target the cell membrane, such as nisin, as mentioned earlier. In addition to targeting lipid II to inhibit cell wall synthesis and thereby exert antibacterial effects, nisin additionally recruits lipid II to form pores through the plasma membrane [70]. Some AMPs exert antimicrobial effects by stimulating cells to produce reactive oxygen species and damaging the membrane respiratory chain, such as microcin J25 (MccJ25) [109,110].

### 3.3. AMPs Acting on Intracellular Targets

AMPs can not only act on the cell wall and cell membrane but also enter the cell through direct penetration or endocytosis [111] and exert anti-microbial effects by targeting the nucleus, organelles, present in fungi, or intracellular proteins [67] (Figure 1). The transmembrane mechanisms of some AMPs are not fully understood. Studies have shown that AMPs can directly cross the cell membranes through the formation of transient toroidal gaps, or directly translocate through membrane boundary defects, and can also transmembrane through receptor-mediated transport pathways. The detailed translocation mechanisms of AMPs are introduced in reference [111]. The following section briefly introduces the intracellular targets and representative AMPs.

(1) The first mechanism is binding to nucleic acids, destroying the conformation of nucleic acids, and inhibiting the synthesis of DNA, RNA, or protein. Histone-derived AMPs have high binding affinity for both DNA and RNA, as they share their entire sequence with a portion of the histone core subunit [112]. Buforin II, a 21-amino-acid peptide with potent antimicrobial activity against a broad range of microorganisms, is a good example of a histone-derived AMP. Buforin II can cause cell death by dissolving cell membranes. However, it has been shown that at low concentrations below MIC, Buforin II can penetrate cell membranes and inhibit cellular functions by binding to the DNA and RNA of cells, resulting in rapid cell death [113]. Indolicidin, a 13-residue AMP isolated from cytoplasmic granules of bovine neutrophils, exhibits activity against Gram-positive and Gram-negative bacteria as well as fungi [114]. Indolicidin can penetrate the bacterial cell membrane, enter the cytoplasm and bind with DNA, inhibit DNA biosynthesis and play a bactericidal role [114]. KW4, a synthetic peptide with lysine and tryptophan repeats, can inhibit cellular function by binding to RNA and DNA, leading to the eradication of *C. albicans* [115]. PR-39, an AMP isolated from the upper half of the pig’s small intestine, kills bacteria by preventing the synthesis of proteins and DNA and causing the degradation of these components [116]. Other AMPs that can interact with nucleic acids include human neutrophil peptide (HNP)-1 [117], porcine β-defensin 2 (pBD2) [118,119] and rondonin [120];

(2) The second mechanism for the inhibition of enzyme/protein activity in nucleic acid and protein synthesis metabolic pathways. In addition to direct DNA binding, indolicidin inhibits DNA relaxation by inactivating DNA topoisomerase I [121]. Microcin B17 (MccB17) is an AMP produced by *E. coli* strains containing the plasmid-borne mccB17 operon. Targeting bacterial DNA gyrase stabilizes the transient DNA gyrase-DNA cleavage complex, interrupting DNA synthesis [122]. MccJ25 is a circular AMP that inhibits RNA polymerase, thereby temporarily terminating the elongation of the transcript [123,124]. Polyphemusin-I, a marine AMP isolated from hemocytes of an American horseshoe crab, possesses potent antimicrobial activities. Screening of intracellular protein targets by an *E. coli* proteome microarray revealed the mechanism by which polyphemusin-I targets nucleic acid-related proteins [43];

(3) A third mechanism is the destruction of nucleic acid damage repair pathways. HNP1 was originally found to penetrate the inner and outer membranes of *E. coli* and inhibit bacterial DNA, RNA and protein synthesis [87,125]. A recent study found that recombinant HNP-1 produced by *E. coli* triggers bacterial apoptosis and that HNP-1 disrupts the DNA damage response pathway by interfering with the binding of RecA to single-stranded DNA (ssDNA) and promotes programmed bacterial death [126];

(4) A fourth mechanism is activity against the ribosome to inhibit protein synthesis. Proline-rich antimicrobial peptides (PrAMPs) were first isolated from mammalian and insect cells as host defense peptides against Gram-negative bacteria [80]. The PrAMPs were divided into two classes based on the conformation in which they bind to the ribosome [127]. Class I PrAMPs, such as Bac7, Onc112, pyrrhocoricin, and metalnikowin, interfere with the initial step of translation to block the peptide transferase center and the peptide exit channel of the ribosome [128], whereas class II PrAMPs, such as apidaecin 1b and Api137, act during translation termination and inhibit protein synthesis by trapping release factors on the 70S ribosome following hydrolysis of the nascent polypeptide chain [129]. There are quite a few recent studies on PrAMPs, which attempt to enhance the antibacterial activity of PrAMPs against bacteria, even drug-resistant bacteria, through de novo synthesis, chemical modification and other methods [130,131,132]. A recent study showed that the PrAMP dimers produced by the bifunctional linker significantly enhanced their antibacterial and anti-biofilm activities against a variety of Gram-negative bacilli, including drug-resistant *Acinetobacter baumannii* strains [132]. In addition, PrAMP dimers have potent immunomodulatory activities and neutralize inflammation through nitric oxide production in macrophages [132].

(5) A fifth mechanism is interference with the proper folding and assembly of proteins. Another intracellular target of PrAMPs is the bacterial heat shock protein DnaK. Inhibition of DnaK leads to protein misfolding and aggregation, which ultimately leads to bacterial death [133]. Pyrrhocoricin, drosocin and apidaecin, representatives of the short PrAMP family, induce permanent closure of the DnaK peptide-binding cavity to inhibit chaperone-assisted protein folding [134].

(6) A final mechanism is inhibition of cell division and blockade of the cell cycle. Temporin L impairs *E. coli* cell division by interacting with FtsZ, a tubular protein with GTPase activity involved in a key step of Z-loop formation at the onset of the division process, and the divisome complex [135]. Temporin L inhibits the enzyme activity and polymeric activity of proteins by binding to FtsZ [136]. C18G can inhibit cell division by stimulating the PhoQ/PhoP two-component signaling system, which regulates QueE transcription and increases the expression of QueE, an enzyme involved in the biosynthesis of a hypermodified guanosine (queuosine) found in certain tRNAs [137].

### 3.4. AMPs Acting on Biofilms

The formation of biofilms is an orderly process that includes four processes: adhesion, sessile growth, maturation and dispersal [2]. The main components of biofilms are polysaccharides, extracellular DNA (eDNA), proteins and other substances secreted by attached cells, which can protect microorganisms in biofilms (more common in chronic bacterial and fungal infections) from adverse environmental conditions and increase the resistance of microorganisms to antibiotics [2]. AMPs can combat biofilms through different mechanisms. In addition to destroying the integrity and stability of cell membranes by penetrating biofilms, interacting with intracellular targets, interfering with cell metabolism, and inhibiting the biosynthesis of nucleic acids or proteins [6,138,139,140], some other antibiofilm mechanisms have also been reported.

(1) The first mechanism is inhibition of the bacterial quorum sensing system (QS). QS is a communication system between bacteria, and it relies on the signal molecules produced by bacteria to function. Preventing biofilm formation by inhibiting QS is one of the important mechanisms of AMPs against biofilms. Subinhibitory concentrations of LL-37 inhibit biofilm formation and affect established *P. aeruginosa* biofilms [141]. LL-37 leads to the downregulation of genes essential for biofilm development by affecting two major QS systems (Las and Rhl) [141]. D- and L-LL-37 significantly downregulated the expression of the Rhl QS-related genes rhlA and rhlB in *P. aeruginosa*, inhibited the synthesis of rhamnosyltransferase, reduced biofilm formation, and degraded an existing *P. aeruginosa* biofilm [142]. LL-37 can also form supramolecular associations with Pseudomonas quinolone signal (PQS) molecules, another important component of the QS regulatory network of *P. aeruginosa*, and the QS signal molecules captured by the peptides are sequestered in coassemblies, thus helping to prevent and eradicate bacterial infection [143]. Gj-CATH2 has strong bactericidal and antibiofilm effects on *Streptococcus mutans*. One of its mechanisms of action is to inhibit the expression of QS systems (luxS and comD/E), resulting in decreased EPS synthesis [144];

(2) A second mechanism is inhibition of the adhesion of microbial cells or induction of the dispersal of cell aggregates. LL-37 promotes twitching of *P. aeruginosa* by regulating genes related to flagellar biosynthesis, leading to bacterial movement and reduced bacterial attachment [141]. LL-37 has been shown to stimulate twitching movement in a dose-dependent manner, and twitching was significantly increased even in the presence of a low concentration of the peptide (4 μg/mL) [141]. In *S. aureus*, LL-37- and LL-37-derived peptides have also been shown to inhibit biofilm formation and degrade existing biofilms [145]. Antibiofilm peptide 1037 can directly inhibit biofilm formation by reducing *P. aeruginosa* swimming and swarming motilities, stimulating twitching movement, and inhibiting the expression of multiple genes involved in biofilm formation [146];

(3) A third mechanism is attenuation of the production of extracellular polymer (EPS), reduction of biofilm formation, and destruction or degradation of the biofilm matrix. AMPs mainly inhibit the synthesis of polysaccharides, eDNA and proteins in the extracellular matrix. Hepcidin 20, an AMP extracted from human liver, can reduce the mass of the extracellular matrix and alter the structure of *Staphylococcus epidermidis* biofilms by targeting polysaccharide intracellular adhesin (PIA) [147]. Human β-defensin 3 (HBD3) also has an antibiofilm effect by reducing the expression of the gene encoding PIA in *S. epidermidis* biofilms [148]. The AMP piscidin-3 derived from fish has nucleosidase activity, which can destroy eDNA of *P. aeruginosa* through the coordination of its N terminus with Cu^2+^ [149]. The acyldepsipeptide antibiotic ADEP4 can activate the ClpP protease to degrade proteins to clear *S. aureus* biofilms [150]. P1, TL, (LIN-SB056-1)2-K and Pc-CATH1, Cc-CATH2, Cc-CATH3, etc., have also been shown to disrupt the biofilm structures of different microorganisms [151,152,153,154];

(4) A fourth mechanism is downregulation of the expression of transporters, reducing the formation of biofilms. The peptide Nal-P-113 inhibits the formation of Porphyromonas gingivalis biofilms by inhibiting the synthesis of ABC transporters and ATP-binding proteins [140,155]. ABC transporters can promote cell surface and cell–cell interactions and play a role in the cell adhesion stage of biofilm formation [156]. At low concentrations in the nanomolar range, HBD2 has been found to reduce biofilm formation without reducing the metabolic activity of *P. aeruginosa* and *Acinetobacter baumannii*. The outer membrane protein profile of bacteria treated with HBD2 was altered, with reduced expression of several proteins, accompanied by an increase in bacterial surface roughness. HBD2-induced structural changes interfere with the transport of biofilm precursors into the extracellular space [157].

In addition, some AMPs can act on cellular stress pathways to prevent the formation of biofilms and clear the established biofilms [158,159]. Some AMPs show a synergistic effect when combined with traditional antibiotics via the promotion of antibiotic uptake [60].

### 3.5. Antiviral Mechanism of AMPs

**Figure 2:**
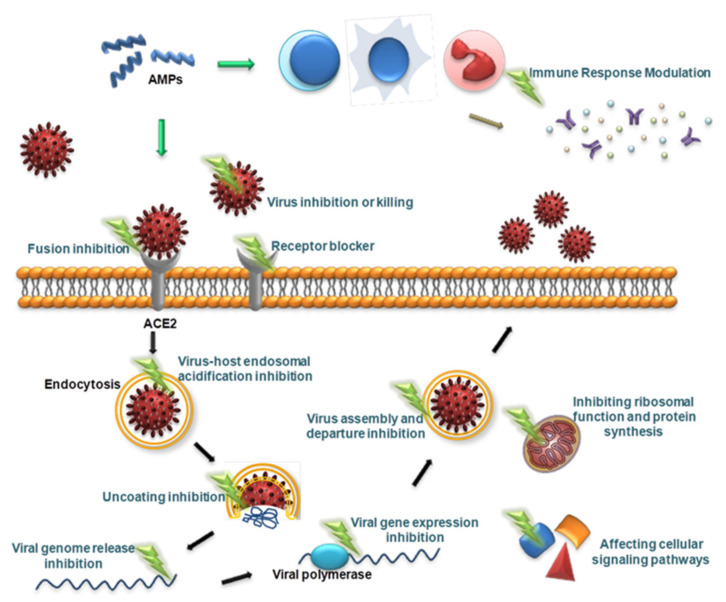
Schematic presentation of the antiviral mechanism of AVPs. AVPs can directly inhibit and kill viral particles, and can also exert antiviral effects at various stages of the viral replication cycle, including adsorption, penetration, uncoating, biosynthesis, assembly, and release. In addition, AVPs can inhibit viral infection by interfering with cellular signaling pathways and modulating the host immune system.

Viruses are different from bacteria, fungi and other microorganisms in that they have the ability to replicate but lack the enzyme system needed to proliferate and can proliferate only in susceptible living cells. They recognize host cells and complete the viral replication cycle within the host cell using low molecular weight substances provided by the host cell [160]. AMPs with antiviral ability are called antiviral peptides (AVPs), and currently, AVPs account for approximately 15% of AMPs [161]. AVPs have become a research hotspot and have shown great potential as medicinal antiviral drugs [162]. AVPs can not only directly inhibit and kill viral particles but also exert antiviral effects at various stages of the viral replication cycle: adsorption, penetration, uncoating, biosynthesis, assembly and release [163] (Figure 2). In addition, AVPs may inhibit viral infection by interfering with cellular signaling pathways and modulating the host immune system [162] (Figure 2). The antiviral mechanisms of AVPs are described below.

(1) The first mechanism is direct inhibition and killing of virus particles. AVPs can disrupt the lipid bilayer in the viral envelope and cause membrane instability, preventing the virus from infecting host cells [160,164]. LL-37 can directly inactivate HSV-1 extracellularly due to its damage to the viral envelope, preventing binding to host cells and infection [165]. The effect of LL-37 directly inducing damage to the viral envelope has also been found for respiratory syncytial virus (RSV), reducing viral binding to and infection of human epithelial cells in vitro. Furthermore, in a mouse model of pulmonary RSV infection, exogenous LL-37 has a protective effect against RSV-mediated disease [166]. In addition, AVPs can accumulate on the envelope surface of the enveloped virus, resulting in membrane tension or the formation of pores that destroy the envelope and release its contents. For example, gloverin protects against the budded viruses of *Autographa californica* multiple nucleopolyhedrovirus by disrupting the virus envelope to form pores [167]. Mucroporin-M1 is a derivative of mucroporin, a cationic HDP from scorpion venom. It has been shown to have antiviral activity against severe acute respiratory syndrome coronavirus (SARS-CoV), influenza A virus (IAV) and measles viruses. Mucroporin-M1 was shown to bind to the virus envelope by surface charge interactions and drastically decrease the infectivity of SARS-CoV, influenza A virus and measles virus. It exerts direct antiviral effects [168]. An extended peptide from bovines, indolicidin, showed a direct inactivation effect on cell-free herpes simplex virus (HSV)-1 virions by targeting the viral membrane/glycoprotein [169];

(2) The second mechanism is blockade of the interaction with cell surface receptors and inhibition of the adsorption and fusion of viruses. LL-37 and engineered LL-37 can inhibit Ebola virus (EBOV) infection. These AMPs target EBOV infection at the endosomal cell-entry step by impairing cathepsin B-mediated processing of EBOV glycoprotein (GP) [170]. Defensins have also been shown to block viral fusion in HIV infection [171]. By monoclonal antibody competition, the regions of interaction with α-defensins were mapped to the D1 domain of CD4 and to a surface contiguous to the CD4- and coreceptor-binding sites of gp120 [172]. The main mechanism of action of HNPs 1–3 against HIV-1 is to prevent HIV-1 from entering cells by interfering with the binding of viral gp120 to CD4+ T cells [172]. HNP-1 can also inhibit HIV-1 replication via the disruption of the protein kinase C (PKC) signaling pathway in an HIV-infected cell [173]. In addition, HNP-1 can inhibit HIV-1 infection after reverse transcription and integration. HNP-1 exerts antiviral effects by affecting more than one step of the HIV replication cycle [173]. Furthermore, AVPs can inhibit viral entry by aggregating virions. AVPs with lectin-like functions have the potential to inhibit viral spread and reduce the viral load by directed copolymerization with viral target proteins and interfering with subsequent dysfunction of that protein [5]. The α-defensins HNP1 and human α-defensin 5 (HD5) have been shown to inhibit BK virus (BKV) infection by targeting early events in the viral life cycle. HD5 can bind to BKV and cause virion aggregation, preventing normal virus binding to the cell surface and uptake by the cell [174];

(3) The third mechanism is inhibition of virus uncoating, virus gene expression and virus assembly and departure processes after entering cells. P9 and P9R, derived from mouse β-defensin-4, showed potent antiviral effects against a variety of respiratory viruses, including influenza virus, SARS-CoV and Middle East respiratory syndrome coronavirus (MERS-CoV). They prevented viral RNA release by inhibiting late endosomal acidification, which is required for uncoating early in the viral life cycle [175,176]. HD5 can inhibit human adenovirus (HAdV) infection at low micromolar concentrations by binding to extracellular HAdV, preventing the escape of internalized virus/defensin complexes from endosomes and thereby preventing infection [177]. The study also revealed that virions strongly colocalize with lysosomes rather than with the nucleus late after infection, suggesting that viral trafficking after infection is altered due to HD5 binding [177]. During HSV-2 infection, the α-defensins HNP2 and HD5 can interact with the DNA of HSV-2, presumably inhibiting HSV-2 infection by blocking gene expression [178]. During IAV infection, in vitro, LL-37 does not prevent viral uptake but rather inhibits IAV replication at a postentry step prior to viral RNA or protein synthesis [179]. During HIV-1 infection, LL-37 directly inhibits HIV-1 reverse transcriptase activity in a dose-dependent manner through protein interactions [180]. Some AVPs act on more than one stage of viral replication. For example, Hp1036 and Hp1239, two newly discovered venom peptides from the scorpion *Heterometrus petersii*, directly interact with the HSV-1 viral membrane extracellularly to inactivate viral particles and, in postinfection steps, inactivate intracellular viral particles and inhibit viral proliferation. They also exhibited potent inhibitory activity during the viral attachment and entry phases [181];

(4) The fourth mechanism is modification of or interference with cellular signaling pathways. Many viruses require the activation of PKC during infection, and AVPs block the viral infection process by inhibiting the activation of PKC [182]. For example, influenza viruses utilize host PKC to regulate ribonucleoprotein complex assembly, a step required for the transition from primary transcription to genome replication during the infection cycle [183]. HNP-1 treatment of influenza virus-infected cells inhibited the activation of PKC and significantly inhibited influenza virus replication and viral protein synthesis [184]. HIV-1 requires phosphorylated PKC for viral fusion, transcription, integration, and aggregation [182,185,186]. HNP-1 acts on primary CD4+ T cells to inhibit HIV-1 replication and block infection by inhibiting PKC phosphorylation [173]. AVPs can also inhibit viral infection by interfering with other signaling pathways. GF-17 and BMAP-18, two cathelicidin-derived AMPs, can directly inactivate Zika virus (ZIKV) and can also inhibit ZIKV infection at higher levels by interfering with the type I interferon signaling pathway. The specific mechanism of action is unclear [187]. Smp76, a scorpion venom peptide, upregulates IFN-β expression by activating interferon-regulated transcription factor 3 phosphorylation, thereby enhancing type I interferon responses and inhibiting Dengue virus and ZIKV infection [188];

(5) The fifth mechanism is the regulation of the host immune response. AMPs can directly bind to various cell surfaces or intracellular receptors on immune cells, triggering the expression of chemokines and cytokines to attract antigen-presenting cells at the site of infection, and then activate immature or nonfunctional T cells, neutrophils, natural killer cells and dendritic cells, eliminating pathogens by inducing humoral and adaptive immunity [189,190,191]. In a viral infection, AVPs can also modulate the host’s immune response. In IAV-infected C57BL/6 mice, genes encoding β-defensin (Defb4), bactericidal permeability-increasing protein (Bpifa1), and cathelicidin (Camp) were differentially regulated after IAV infection, and the expression of Defb4 varied according to different virus strains. For example, β-defensin reduced the infectivity of the A/CA/04/2009 virus but not the A/PR/08/1934 virus. β-Defensin also altered the innate immune cell repertoire, with increased alveolar macrophage and CD103(+) dendritic cell counts and decreased CD11b(+) dendritic cell and neutrophil counts in mice pretreated with β-defensin [192]. AMPs appear to play a dual role in microbial infection, with proinflammatory and immunosuppressive effects [193]. LL-37 had anti-IAV activity and reduced disease severity and viral replication in infected mice. LL-37 protected against influenza virus infection by regulating inflammation in the lungs [191]. H1N1-infected mice treated with LL-37 had lower concentrations of proinflammatory cytokines in the lung than did infected animals that had not been treated with cathelicidin peptides. This finding suggested that AMPs may play a protective role by inhibiting excessive inflammation [191].

(6) AVPs are used as adjuvants to design and synthesize multiepitope vaccines against viruses to enhance the prevention and treatment of the viruses [194,195,196]. For example, in the development of an effective DNA vaccine against bovine herpesvirus 1 (BOHV-1), the fusion of bovine neutrophil β-defensin 3 (BNBD3) with the protective antigen in the DNA vaccine increased the cell-mediated immune response [197]. In the development of universal influenza vaccines that provide protection against all or most influenza subtypes, many researchers have attempted to generate conserved epitopes of influenza virus antigens in the form of peptides with the hope of broadly inducing cross-reactivity against influenza virus infection. Some of the multiepitope peptide vaccine candidates have entered the clinical trial stage, such as Multimeric-001 and FLU-v [198].

AVPs have been found to have anti-coronavirus effects in past studies. Due to the wide spread of severe acute respiratory syndrome coronavirus 2 (SARS-CoV-2) and the lack of effective drugs, AVPs are expected to be a new therapeutic strategy. Much research has been conducted in this field, and the current research focus is on finding and developing AVPs that can disrupt viral cell membranes and block viral entry into cells to reduce the viral load of SARS-CoV-2 entering host cells. The potential AVPs against SARS-CoV-2 and their mechanisms of action are summarized in Table 1.

## 4. Advantages, Disadvantages and Clinical Applications of Antimicrobial Peptides

As mentioned above, AMPs have a wide range of sources, diverse targets, and broad-spectrum antimicrobial, immunomodulatory activities and other host-beneficial activities, such as anticancer or wound healing effects. They can effectively act on multidrug-resistant bacteria, and drug resistance to them is not easily developed, so they have broad application prospects. However, there are a number of problems that prevent their clinical application in humans. Due to the small-molecule nature of AMPs, the direct extraction of natural AMPs from animal and plant tissues is low-yield, time-consuming, complex and expensive, making it impossible to achieve large-scale production. Chemical synthesis and genetic engineering have become the main means to obtain AMPs [211,212,213]. However, chemically synthesized peptides are expensive. Besides, through genetic engineering, the direct expression of AMP genes in microorganisms may cause the host microorganisms to commit suicide and not yield the expression products. Although this shortcoming can be overcome by expressing AMP genes in the form of fusion proteins, there are still few expression products. In addition, many AMPs are easily cleaved by proteases in the human body and are excreted rapidly through the kidneys, resulting in a particularly short half-life. Due to the extremely short circulation time, the antibacterial effect in the body is short-lived, and the antibacterial activity is not ideal [214]. Antibacterial activity may also be lost due to serum binding or inactivation via physiological salt concentrations. These effects greatly limit the use of AMPs. Cytotoxicity and lack of specificity are also important factors preventing the clinical application of AMPs [214].

For better clinical application, researchers have explored different strategies to overcome the current shortcomings of AMPs. AMPs are easily synthesized by automated protein synthesis [215,216]. AMPs are abundantly produced in heterologous expression systems in microbial cells [213,217]. Various chemical modifications, such as d-amino acid insertion [218], cyclization [219], acetylation [220], encapsulation modification [221,222] and synthetic AMPs [223], have been used to optimize candidate AMPs, improve the stability of peptides to proteases, reduce cytotoxicity, and control half-life and release curves [224].

Currently, a few AMPs have been approved by the US Food and Drug Administration (FDA) for clinical treatment. Listed below are several AMPs approved by the FDA. Gramicidin is extracted from *Bacillus brevis* and has an antibacterial effect by targeting the cell membrane. It has an obvious effect on Gram-positive bacteria and has an inhibitory effect on some Gram-negative bacteria at high concentrations [225]. Due to its severe cytotoxicity, its clinical application is greatly limited. It is generally made into an ointment formulation for the prevention and treatment of purulent skin diseases. Polymyxin is an antibacterial polypeptide found in the culture medium of *Paenibacillus polymyxa*. It also targets the cell membrane to exert an antibacterial effect and has a killing effect on most Gram-negative bacilli, including antibiotic-resistant *P. aeruginosa* and *Acinetobacter* [226]. It can be applied locally or systemically. It is mainly used for infections of wounds, the urinary tract, eyes, ears, the trachea and other body parts caused by *P. aeruginosa* and other pseudomonads. It can also be used for sepsis and peritonitis. The main adverse reactions to polymyxin include renal toxicity and neurotoxicity [226], so it is necessary to closely monitor the indicators of related adverse reactions during the application process. Vancomycin is a glycopeptide antibiotic that plays an antibacterial role by inhibiting cell wall synthesis and has a strong antibacterial effect on Gram-positive bacteria. It is usually used systemically and is used for infectious diseases caused by drug-resistant bacteria [227]. In recent years, reports of drug-resistant *S. aureus* have become increasingly frequent, and the active search for alternatives that may prolong the clinical use of this important antibiotic has become a research hotspot. Examples include telavancin, which was approved by the FDA in 2009, and dalbavancin and oritavancin, which were approved by the FDA in 2014 [227]. Daptomycin is a lipopeptide antibiotic produced by *Streptomyces solani* for the treatment of concurrent skin and skin structure infections, such as abscesses, surgical incision infections, and skin ulcers, including those caused by MRSA [227,228]. The mechanism of action of daptomycin is different from that of other antibiotics. By disrupting the transport of amino acids across the cell membrane, daptomycin blocks the biosynthesis of peptidoglycan on the bacterial cell wall and changes the properties of the plasma membrane. In addition, it can also kill bacteria by destroying their cell membranes and causing their contents to leak out [225,228]. There are also examples of antiviral AMPs used in clinical practice. For example, enfuvirtide is a synthetic peptide HIV fusion inhibitor that can bind to viral envelope glycoproteins and prevent the conformational changes necessary for virus fusion with cell membranes, thereby inhibiting HIV-1 replication [229]. The application method is subcutaneous injection for HIV infection, combined with reverse transcriptase inhibitor drugs [229]. Lopinavir is a type of peptidomimetic approved by the FDA for the treatment of HIV and has been shown to work against SARS-CoV-2 in recent clinical trials [230].

Preclinical feasibility studies are already underway for a variety of AMPs, but few have entered clinical trials. Table 2 summarizes some of the AMPs that have entered clinical or preclinical testing. Most AMPs are analogs of natural AMPs, but some are fully synthetic. Additional clinical trial data are needed to improve peptide stability, reduce cytotoxicity, and improve pharmacokinetics and pharmacodynamics when these molecules are used in a clinical setting.

In addition, clinical studies have demonstrated that AMP levels can be used clinically as a prognostic indicator for some diseases. For example, elevated α-defensin levels may increase the risk of thromboembolic complications [242]. In clinical studies, neutrophil-derived HBP levels were significantly increased in COVID-19 patients who developed organ failure compared with those in patients who did not. These results suggest that HBP levels can predict the development of organ dysfunction caused by COVID-19 [243].

## 5. Conclusions

AMPs are unique molecules with a wide range of sources and broad-spectrum antimicrobial activity that can exert antimicrobial effects via multiple mechanisms of action, including targeting cell walls, cell membranes, intracellular components, and biofilms. The multiple mechanisms of action lead to a low possibility of microbial resistance development and indicate broad application prospects. In addition, AMPs also have antiviral activity and can act on each stage of viral replication. Due to the COVID-19 pandemic, researchers are devoted to finding AMP markers that can help to judge the severity of COVID-19, and research on AMPs against SARS-CoV-2 is increasing, but further research is needed prior to clinical application. Although AMPs have many advantages, there are still many disadvantages, such as instability, poor bioavailability, short half-life, cytotoxicity and lack of specificity, which limit their clinical application. Abundant research is being directed to this area, including analyzing the relationship between the structure and activity of AMPs, modification of AMPs, artificial synthesis of AMPs, identification of a suitable system for commercial production, and clinical research on AMP drugs. It is believed that in the near future, these problems will be overcome and that AMPs will be safely and effectively used in clinical practice for the benefit of humankind.

## Figures and Tables

**Table 1:** Potential antiviral peptides against SARS-CoV-2 and their mechanisms of action.

AMP	Source	Peptide Type	Sequence	Infection Model	Effect and Mechanism of Action	Reference
HD5	Human intestinal Paneth cells	β-sheet	ATCYCRTGRCARESLSGVCEISGRLYRLCCR	in vitro	Shields ACE2 from binding to SARS-CoV-2	[199]
P9R	Modification	β-sheet	NGAICWGPCPTAFRQIGNCGRFRVRCCRIR	in vitro	Binds to the virus and inhibits virus–host endosomal acidification	[176]
Brilacidin	Synthetic	Peptidomimetic	Not provided	in vitro	Interferes with virus entry and destroys virus integrity; synergistic antiviral activity when combined with remdesivir	[200]
Nisin H	Lactic acid bacteria	Cyclic peptide	FTSISMCTPGCKTGACMTCNYKTATCHCSIKVSK	in vitro	Competes with SARS-CoV-2 for binding to hACE2	[201]
Caerin 1.6 and caerin 1.10	Amphibian	α-helical	GLFSVLGAVAKHVLPHVVPVIAEK/GLLSVLGSVAKHVLPHVVPVIAEKL	in silico discovery	Interacts with Arg995 located in the S2 subunit of Sgp, which is the key subunit that plays an essential role in viral fusion and entry into the host cell through ACE2	[202]
DP7	Synthetic	Not provided	VQWRIRVAVIRK	in vitro	Inhibits SARS-CoV-2 S protein-mediated cell fusion and inhibits SARS-CoV-2 3CLpro enzyme activity	[203]
Peptoid 1 and its derivatives	Synthetic	α-helical	Not provided	in vitro	Inactivates enveloped viruses through a membrane disruption mechanism	[165]
LL-37	Human	α-helical	LLGDFFRKSKEKIGKEFKRIVQRIKDFLRNLVPRTES	in vitro and in vivo	Simultaneously blocks viral S1 and cloaks ACE2	[204]
HBD2	Human mucosal epithelium	β-sheet	GIGDPVTCLKSGAICHPVFCPRRYKQIGTCGLPGTKCCKKP	in vitro	Binds the SARS-CoV-2 RBD and blocks viral entry	[205]
Meucin-18 and its derivative	Venom scorpion	α-helical	FFGHLFKLATKIIPSLFQ/FFGHLFKLTTKIIPSLFQ	in vitro	Interacts with the RBD of the spike protein of SARS-CoV-2 to inhibit the spike protein’s interaction with the ACE2 receptor	[206]
Plectasin	*Pseudoplectania nigrella*	Not provided	GFGCNGPWDEDDMQCHNHCKSIKGYKGGYCAKGGFVCKCY	in silico discovery	Interacts with the nucleocapsid of coronaviruses	[207]
HNP1	Human neutrophil	β-sheet	Not provided	in vitro	Destabilizes and precipitates spike protein and inhibits the interaction of spike with the ACE2 receptor	[208]
RC-101	Modification	Not provided	Not provided	in vitro	Destabilizes and precipitates spike protein and inhibits the interaction of spike with the ACE2 receptor	[208]
RTD-1	Rhesus macaque leukocytes	Cyclic peptide	GFCRCLCRRGVCRCICTR	in silico discovery	Modulates host immunity by inhibiting the release of proinflammatory cytokines, protecting the body from immune-mediated organ damage	[209,210]

**Table 2:** AMPs in clinical or preclinical trials.

AMP	Template	Phase of Clinical Trials	Administration	Application	Reference
Iseganan	Protegrin-1	Phase 2/3	Topical	Prevention of ventilator-associated pneumonia	[231]
XF-73	Porphyrin	Phase 1	Nasal gel	Prevention of postoperative *S. aureus* colonization and infection	[232]
P-113	Histatin 5	Phase 2	Mouth rinse	Reduce gum bleeding, gingivitis and plaque	[233]
Omiganan	Indolicidin	Phase 2	Topical gel	Treatment of mild to moderate atopic dermatitis	[234]
LTX-109	Synthetic peptidomimetic	Phase 1/2	Topical	Prevention of nasal infections caused by methicillin-sensitive/resistant *S. aureus*	[235]
Onc72	Oncocin	Preclinical	Subcutaneous	Treatment of antibiotic-susceptible *K. pneumoniae*	[236]
OP-145	LL-37	Preclinical	Implant coating	Prevention of *S. aureus*-induced biomaterial-associated infections	[237]
Lactoferrin	Not applicable	Phase 4	Oral	Prevention of neonatal sepsis	[238]
Murepavadin	Protegrin-1	Phase 1	Intravenous	Treatment of pneumonia caused by *P. aeruginosa* infection	[239]
Surotomycin	Daptomycin	Phase 2	Oral	Treatment of *C. difficile*-associated infection	[240]
LL-37	Not applicable	Phase 2	Topical	Control of infection of diabetic foot ulcers	[241]

## Data Availability

Not applicable.
